# Disseminated Tuberculosis of the Lungs, Pleura, Mediastinal Lymph Nodes, and Pericardium: A Rare Case Report

**DOI:** 10.7759/cureus.45562

**Published:** 2023-09-19

**Authors:** Sankalp Yadav

**Affiliations:** 1 Medicine, Shri Madan Lal Khurana Chest Clinic, Moti Nagar, New Delhi, IND

**Keywords:** left sided pleural effusion, pleural effusion, pericardial effusion, mediastinal lymphadenopathy, pleura, lungs, cbnaat/ xpert/ rif assay, mtb (mycobacterium tuberculosis), disseminated tuberculosis

## Abstract

Tuberculosis is mainly known to affect the lungs, but it can manifest at various extrapulmonary sites. Disseminated tuberculosis is a relatively rare clinical condition, and cases with no history of the disease are sparse. A case of an 18-year-old Indian male is presented. He came with complaints of chest pain, coughing with expectoration, and loss of appetite. The diagnostic workup led to a definite diagnosis of disseminated tuberculosis with involvement of the lungs, pleura, mediastinal lymph nodes, and pericardium. He was initiated on a fixed-dose anti-tubercular treatment per the national guidelines.

## Introduction

Tuberculosis is a highly prevalent infectious disease with a very high morbidity and mortality rate [1]. This disease can manifest in either pulmonary or extrapulmonary forms [2]. Pulmonary involvement is commonly reported, but extrapulmonary tuberculosis constitutes about 10-15% of cases [2]. The Global Tuberculosis Report by the WHO reports an incidence of 188 and a prevalence of 312 per one lakh (0.1 million) population in India [1].

Disseminated tuberculosis is a relatively rare form of tuberculosis that is defined as the involvement of two or more non-contiguous sites due to hematogenous dissemination of *Mycobacterium tuberculosis*, occurring because of a progressive primary infection, reactivation of a latent bacterial focus with subsequent spread, or rarely from an iatrogenic origin [3,4]. Its prevalence in immunocompetent adults is estimated at less than 2% of all cases of tuberculosis and approximately 20% of total extrapulmonary tuberculosis cases [5].

Herein, a case of a young Indian male who presented with chest pain associated with coughing, expectoration, and loss of appetite is presented. The diagnosis of disseminated tuberculosis was difficult due to the absence of a history of disease or any contact with simultaneous involvement of multiple sites in the body. A diagnosis was achieved with a cartridge-based nucleic acid amplification test of the sputum, diagnostic pleural tapping with pericardiocentesis, and a biopsy of the mediastinal lymph nodes. He was put on anti-tubercular chemotherapy for his weight.

## Case presentation

An 18-year-old Indian non-diabetic male belonging to a low socioeconomic group reported complaints of chest pain, cough with expectoration, and loss of appetite. He was alright 20 days ago when he had chest pain; it was generalized and not associated with any aggravating or relieving factors. The pain was localized to the right side, progressed from off and on to continuous, and was dull in nature. It was not associated with shortness of breath, cold sweats, nausea, or vomiting. He also had a cough with expectoration for two weeks. The cough was persistent and associated with a yellow-colored, non-foul-smelling, non-blood-tinged expectoration. Besides, he had a loss of appetite for nearly 18 days. There was no history of seizures, night sweats, fever, or weight loss. Additionally, there was no history of tuberculosis in him or any of his contacts.

Further, he was a non-drinker and had never used drugs before. There was no prior history of stays at night shelters, refugee camps, or detention.

On general examination, he was a lean, hemodynamically stable individual with no pallor, cyanosis, icterus, clubbing, or edema. The systemic examination was remarkable for decreased breath sounds on the left hemithorax with crackles on auscultation and inspiratory rales on the right hemithorax. He underwent a detailed diagnostic workup, as mentioned in Table 1.

**Table 1:** Diagnostic workup of the patient HGB: Hemoglobin; MCH: Mean Corpuscular Hemoglobin; MCHC: Mean Corpuscular Hemoglobin Concentration; MCV: Mean Corpuscular Volume; PCV: Packed cell volume; RDW: Red Cell Distribution Width; RBC: Red Blood Cell; WBC: White Blood Cell; DLC: Differential Leukocyte Count; ESR: Erythrocyte Sedimentation Rate; ALK PHOS: Alkaline Phosphatase; AST: Aspartate Aminotransferase; ALT: Alanine Aminotransferase; HCV: Hepatitis C Virus; USG: Ultrasonography

Investigation	Results	Reference range
HGB	10.8	11.5-16.0 g/dL
MCH	22.7	27-33 pcg
MCHC	32.1	31-36 g/dL
MCV	70.9	85-100 fl
PCV	40.0	38.3% to 48.6%
RDW	12.0	0-14%
RBC	4.9	4.7 to 6.1 million cells/mcL
WBC	7.0	4.5-12.0 K/uL
DLC		
Neutrophils	65	55-70%
Lymphocytes	25	20-40%
Monocytes	6	2-8%
Eosinophils	2	1-4%
Basophils	2	0-1%
ESR	56.0	0 to 22 mm/hr
Serum sodium	135.0	135-145 mmol/L
Serum potassium	4.65	3.5-5.1 mmol/L
Serum calcium	8.7	8.5-10.5mmol/L
Serum chloride	99.8	98-107 mmol/L
Blood culture	Sterile	Sterile
Serum bilirubin (total)	0.20	0.2-1.0 mg/dL
Serum bilirubin (direct)	0.30	0.2-1.0 mg/dL
Serum bilirubin (indirect)	0.24	0.2-1.0 mg/dL
ALK PHOS	111.0	30-115u/L
Albumin	3.6	3.5-5 g/dl
Serum creatinine	0.58	0.51-0.95 mg/dL
AST	23.0	0-40u/L
ALT	22.0	0-40u/L
Anti-HCV antibodies	Non-reactive	Reactive-Non-reactive
HIV (I and II)	Non-reactive	Reactive-Non-reactive
Fasting blood sugar	91.0	70-99 mg/dL
Activated partial thromboplastin time	33	25-35 seconds
Serum angiotensin-converting enzyme levels	30	<40 nmol/mL/min.
C-reactive proteins	0.3	0.3 to 1.0 mg/dL
Bone marrow biopsy	Unremarkable	Unremarkable-Remarkable
Mantoux test	22	0-15 millimetres
Echocardiography	Unremarkable	Unremarkable-Remarkable
Electrocardiogram	Unremarkable	Unremarkable-Remarkable
USG-whole abdomen	Unremarkable	Unremarkable-Remarkable

**Figure 1:**
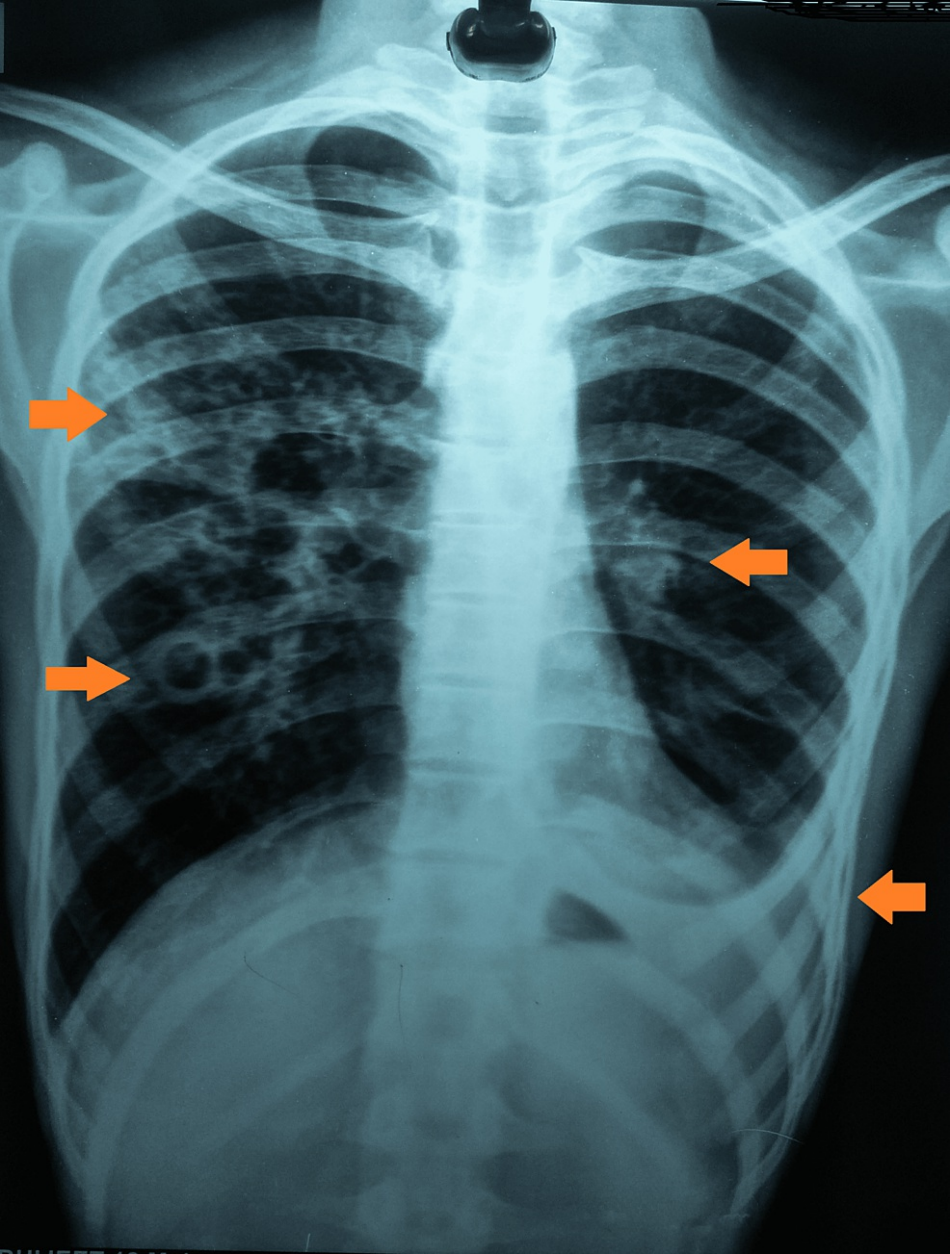
Chest radiograph (P-A) view showing bilateral involvement of the lungs P-A: Posteroanterior

A chest radiograph was done, which was suggestive of multiple cavitary lesions with consolidation of the right upper lobe and a left pleural effusion (Figure 1).

**Figure 2:**
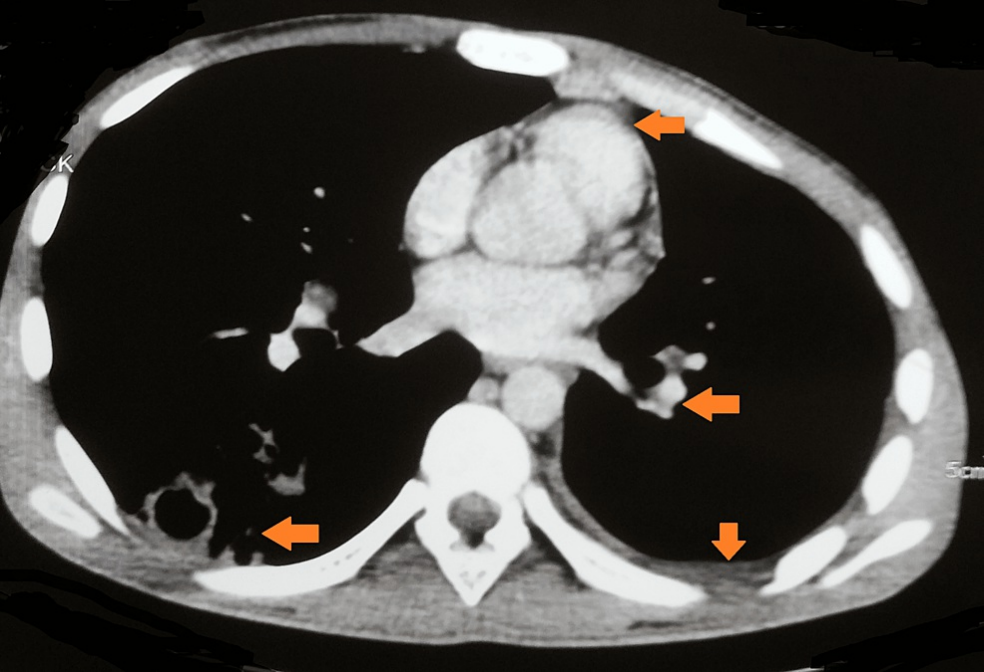
CT chest suggestive of pleural effusion, pericardial effusion, mediastinal lymphadenopathy, and pleural cavities with consolidation CT: computed tomography

**Figure 3:**
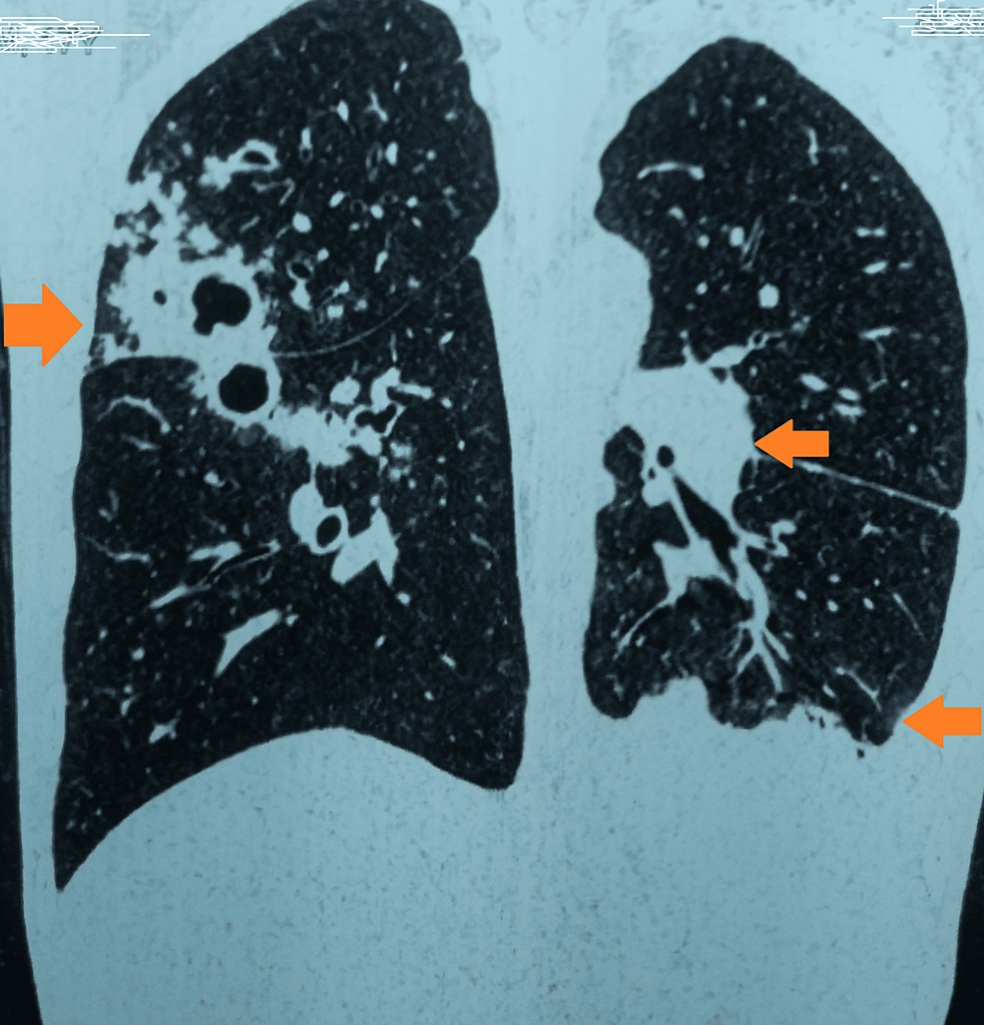
CT thorax showing bilateral involvement of the lungs CT: computed tomography

A computed tomography of the thorax was suggestive of multiple patchy areas of subsegmental consolidation with internal areas of cavitation in the anterior and posterior segments of the right upper lobe, the apicoposterior segment of the left upper lobe, the superior and medial, posterior, and anterior basal segments of the right lower lobe and the anterior basal segments of the left lower lobe. Multiple nodules in the tree's bud appearance were seen. There was mild left pleural and pericardial effusion with a mild loss of left lung volume. There were multiple enlarged heterogeneously enhancing necrotic lymph nodes noted in the right paratracheal (18 mm), anterior-posterior window (12 mm), prevasuclar (19 mm), subcarinal (23 mm), right hilar (16 mm), and left hilar (20 mm) locations (Figures 2, 3).

A sputum smear for acid-fast bacilli was negative, but a cartridge-based nucleic acid amplification test of the sputum detected *Mycobacterium tuberculosis* (low) with no resistance to rifampicin. A line-probe assay was also positive for *Mycobacterium tuberculosis*, with no resistance to rifampicin or isoniazid. A culture of sputum was negative. He underwent a thoracentesis and a pericardiocentesis; the results are tabulated in Table 2 and Table 3.

**Table 2:** Results of thoracentesis ADA: Adenosine deaminase; CBNAAT: Cartridge-based nucleic acid amplification test

Test	Result	Reference range
Physical appearance	Straw colored	Colorless
Protein	6.2	1-2 g/dL
Glucose	20 mg/dl	74-106 mg/dL
pH	6.8	7.60-7.64
Cells	90% Lymphocytes	75% macrophages
ADA	61	<30 U/L
CBNAAT	Negative	Negative-Positive
Culture	Negative	Negative-Positive

**Table 3:** Results of pericardiocentesis ADA: Adenosine deaminase; CBNAAT: Cartridge-based nucleic acid amplification

Test	Result	Reference range
Physical appearance	Cloudy	Colorless
Protein	5.1	1-2 g/dL
Glucose	28 mg/dl	74-106 mg/dL
Gram stain	Negative	Negative-Positive
Cells	91% Lymphocytes	75% macrophages
ADA	61	0-40 U/L
CBNAAT	Negative	Negative-Positive
Culture	Negative	Negative-Positive

Further, a fiber optic bronchoscopy was performed, and the tests from the specimen, including the acid-fast bacilli smear, culture, and malignant cells, were negative. The fluid from the bronchoalveolar lavage had a cluster of differentiation 4/cluster of differentiation 8 (CD4/CD8) ratio of 1.7. Serum angiotensin-converting enzyme levels were 92 U/L. The likelihood of a lymphoma was taken into consideration based on the clinical and radiological findings. Hence, a bone marrow aspiration was done, which showed a reactive marrow, and a biopsy was negative for granulomas or abnormal cells. The culture of bone marrow for mycobacterial species produced negative results. A mediastinoscopy-guided lymph node biopsy was undertaken, and the histopathological examination showed epithelioid cell granulomas with Langhans giant cells in a necrotic background consistent with tuberculosis. A cartridge-based nucleic acid amplification test of the biopsied sample detected *Mycobacterium tuberculosis *(low) with no resistance to rifampicin. Further, a culture of the same was negative.

He was initiated on a fixed-dose combination of anti-tubercular drugs with isoniazid 300 mg, ethambutol 1000 mg, rifampicin 450 mg, and pyrazinamide 1500 mg in the initiation phase of two months and four months of the continuation phase with three drugs, i.e., isoniazid 300 mg, rifampicin 450 mg, and ethambutol 1000 mg. He did well initially for a month with no adverse drug reactions and gained weight (about 2 kg), but later he requested a transfer out to his native place, and his request was acknowledged. He was counseled for treatment adherence and a high-protein diet, with a regular follow-up at the nearest infectious diseases clinic.

## Discussion

Disseminated tuberculosis poses a serious risk to health, particularly if there is delay in diagnosis and management [3]. Due to the nonspecific clinical picture, the diagnosis is challenging. Additionally, there are few tools available for laboratory diagnosis; in addition, the acid-fast bacilli smear has a low sensitivity, cultures take a long time, and chest radiographs cannot readily detect miliary changes. Furthermore, this disease has a subtle and ambiguous clinical presentation [6].

It is a significant contributor to morbidity and mortality in almost all age groups [6]. Multiple factors have been reported to be associated with the development of disseminated tuberculosis, including human immunodeficiency virus infection or acquired immunodeficiency syndrome (AIDS), with or without added factors of immunosuppression such as the use of biologicals and immunosuppressive drugs for the treatment of various medical disorders, alcoholism, the increasing prevalence of organ transplantation, chronic hemodialysis, chronic liver disease, malignancies, diabetes mellitus, and silicosis [6].

Khan et al. reported that there was a diagnostic delay of nearly one month in disseminated tuberculosis [3]. This is mostly attributed to non-specific clinical features [3]. The diagnosis of disseminated tuberculosis is even more challenging in children due to vague clinical presentations, often resulting in missed diagnoses [3-6]. The disease presents differently in adults and children. In adults, the commonest clinical features are anorexia, night sweats, abdominal pain, fatigue, hemoptysis, headache, dyspnea, fever, mental changes, ascites, pleural effusion, and lymphadenopathy. In children, diarrhea, vomiting, seizures, hepatomegaly, splenomegaly, jaundice, and meningism are frequently reported [6].

The gold standard of diagnosis is the culture of the bacteria from the specimen obtained from the infected organ [7]. Although it takes a significant amount of time to get the result, which could impact the outcome of management [7], Besides, a clinical correlation with radiometric findings and histology is helpful in the timely initiation of management [6]. Also, reports of measuring biomarkers such as adenosine deaminase and interferon gamma in the supernatant of fluid specimens like pleural fluid, ascites, pericardial fluid, and cerebrospinal fluid are available but are still in their early stages and not included in the diagnostic tools [6].

There is no consensus on the total duration of treatment [6]. However, the national guidelines recommend treatment for six months. After the completion of six months, the extension is based on improvements in clinical condition and drug susceptibility pattern. Management is conservative with anti-tubercular drugs. However, excision of large, swollen lymph nodes is recommended [8].

Even after advancements in the management of patients with disseminated tuberculosis, there is a high mortality rate ranging between 25 and 30% [6]. The documented mortality predictors are meningismus, liver cirrhosis, leukopenia, advancing age, presence of underlying disease, leukocytosis, altered mental status, and night sweats [3,5,6,9].

In a 10-year retrospective study by Wang et al. on 3058 culture-confirmed tuberculosis patients, only 5.4%, i.e., 164, had disseminated tuberculosis. Further, they reported a mortality rate of 31.1% [10]. Another retrospective study done over three decades in India reported the commonest involved organ was the lungs (90.1%), followed by the brain (73.3%), liver (72%), spleen (44%), kidneys (37%), bone marrow (17%), adrenals (12.2%), intestines (11.4%), pancreas (8.5%), and reproductive organs (6.9%). Additionally, they stated that in 25% of instances, an ante-mortem tuberculosis diagnosis was not suspected [11].

A case of disseminated tuberculosis involving four different sites in a young Indian male is presented. There is a paucity of data on such presentations of disseminated tuberculosis, and this case emphasizes the need for reporting similar presentations from endemic countries. However, this was only one case, and large-scale studies are required in disseminated tuberculosis to modify the existing management algorithm.

## Conclusions

An 18-year-old male was diagnosed on the basis of a cartridge-based nucleic acid amplification test of the sputum, a biopsy of the mediastinal lymph node, and a culture of the sputum, backed by radiometric investigations with thoracentesis and paricardocentesis. He was initiated on anti-tubercular treatment for one year. Disseminated tuberculosis is a challenging diagnosis, and therefore it requires a great degree of clinical suspicion and prompt use of diagnostic techniques to achieve favorable outcomes.
